# Ultrasonic-Assisted Extraction of Astaxanthin from Shrimp By-Products Using Vegetable Oils

**DOI:** 10.3390/md21090467

**Published:** 2023-08-25

**Authors:** Ioannis Panagiotakopoulos, Haralabos C. Karantonis, Ioannis Geraris Kartelias, Constantina Nasopoulou

**Affiliations:** Laboratory of Food Chemistry and Technology and Quality of Food of Animal Origin, Department of Food Science and Nutrition, School of Environment, University of Aegean, Metropolitan Ioakeim 2, 81400 Lemnos, Greece; panagiotakopoulos.ioannes@gmail.com (I.P.); chkarantonis@aegean.gr (H.C.K.); jkartel@otenet.gr (I.G.K.)

**Keywords:** shrimp by-products, astaxanthin, ultrasound-assisted extraction (UAE), olive oil (OO), radical scavenging activity, accelerated study, encapsulation efficiency

## Abstract

Background: The use of conventional astaxanthin extraction methods, typically involving organic solvents, leads to a heightened environmental impact. The aim of this study was to explore the potential use of environmentally friendly extraction solvents, such as vegetable oils, for recovering the shrimp by-product astaxanthin. Methods: Ultrasound-assisted extraction (UAE) in vegetable oils, including olive oil (OO), sunflower oil (SO), and flaxseed oil (FO), was employed to extract astaxanthin. The astaxanthin antioxidant activity was evaluated using an ABTS assay, and a mixture of gum Arabic and soy lecithin was used to form coacervates to produce astaxanthin encapsulation. Results: A by-product–vegetable oil ratio of 1:60, extraction time of 210 min, 60% amplitude of the extraction process, and the use of OO as the extracting medium resulted in an astaxanthin yield of 235 ± 4.07 μg astaxanthin/g by-products. The astaxanthin encapsulation efficiency on day 0 and astaxanthin recovery on day 1 were recorded at 66.6 ± 2.7% and 94.4 ± 4.6%, respectively. Conclusions: The utilization of OO as an extraction solvent for astaxanthin from shrimp by-products in UAE represents a novel and promising approach to reducing the environmental impact of shrimp by-products. The effective astaxanthin encapsulation efficiency highlights its potential application in food industries.

## 1. Introduction

Shrimp, which is one of the primary subclasses of crustaceans, is available on the market fresh or processed. The shrimp head, the carapace, and the shell are generated as processing byproducts in both headless and peeled processed shrimp and may account for up to 50–60% of total body weight [1,2]. These byproducts are a possible source of food additives because the cephalothorax contains valuable bioactive components with high contents of omega-3 polyunsaturated fatty acids (PUFAs), mostly docosahexaenoic and eicosapentaenoic acids. These long-chain PUFAs are well-known for their great health advantages [3]. Shrimp shells also contain protein, minerals, and carotenoids, such as astaxanthin, which is a powerful antioxidant pigment [4]; therefore, they could contribute added value to functional food products.

**Figure 1:**
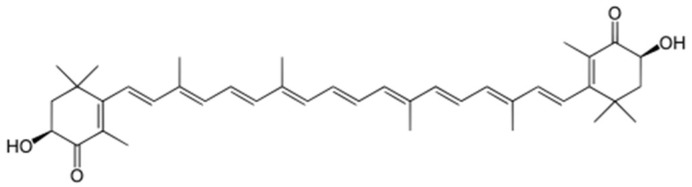
Chemical structure of astaxanthin. Astaxanthin has potent antioxidant ability and is the most abundant carotenoid in crustaceans (75–95% of total pigment), giving them their reddish-orange color. Astaxanthin has a tenfold more potent antioxidant capacity than other carotenoids, such as zeaxanthin, lutein, and canthaxanthin, and 100 times increased antioxidant ability compared with a-tocopherol (vitamin E) and ascorbic acid (vitamin C) [5,6,7] due to the presence of hydroxyl and ketonic functional groups in its structure [8,9].

Astaxanthin (3,3′-dihydroxy-beta, beta-carotene-4,4′-dione, Figure 1) is a xanthophyll carotenoid and is categorized as a lipophilic carotenoid.

Astaxanthin has a protective effect against oxidative damage via a variety of mechanisms, including singlet oxygen neutralization, radical scavenging to prevent chain reactions, membrane structure preservation via inhibition of lipid peroxidation, immune system function enhancement, and gene expression regulation [10,11]. Among other health benefits, astaxanthin possesses anticancer activity, a protective effect against cardiovascular diseases, anti-diabetic capacity, and anti-inflammation ability [11,12].

Several methods can be used to extract astaxanthin, such as the conventional chemical method with organic solvents, the alkaline method, or maceration. Conventional extraction methods are typically more time-consuming, require an increased amount of solvent to enhance the extraction process, and result in chemical degradation [13,14]. Carotenoid components from crustaceans can also be extracted utilizing vegetable oils as solvents [1,15,16,17,18]. Vegetable oils have the benefit of being an effective barrier to oxygen, which diminishes the oxidation process [15]. An alternative method of carotenoid compound extraction from shrimp by-products is the combination of ultrasound-assisted extraction (UAE) using novel green solvents, such as vegetable oils. UAE is a quick and effective method of extraction. The effect of acoustic cavitations generated within the solvent by the passage of an ultrasonic wave is primarily responsible for the improved extraction efficiency. This effect could be attributed to the mechanical impact of ultrasound on the food matrix due to the high shear force resulting from the cavitation bubbles of ultrasonic waves, increasing the contact area between the liquid and solid phase, and therefore permitting greater penetration of the solvent into the food tissue. Consequently, the solute diffuses rapidly from the solid phase to the solvent. As a result, UAE has been widely used in the extraction of different natural compounds [16,19,20].

As a highly unsaturated compound, astaxanthin is vulnerable to deterioration (oxidation) in the presence of extremes in temperature, oxygen level, light, and pH. The degradation of carotenoids affects the food’s nutritional value, color, and organoleptic qualities, in addition to its loss of functional attributes [21]. Encapsulation may help to overcome problems associated with the molecule’s degradation since it is a process by which molecules or materials are enclosed within a protective shell or coating, often referred to as a capsule or microcapsule. The encapsulation technique provides physical and chemical protection. The encapsulating shell acts as a physical barrier, shielding the encapsulated molecules from external factors such as oxygen, moisture, light, and temperature variations. These environmental factors can cause degradation, chemical reactions, or loss of functionality in certain molecules. Encapsulation can also offer chemical protection by isolating the encapsulated molecules from reactive substances present in the surrounding environment. For example, encapsulating sensitive molecules can prevent their interaction with other compounds, preserving their stability and effectiveness [22]. Various methods were researched to increase the application of AST in functional food products, such as structural modification; microencapsulation; and integration into liposomes, emulsions, and nanoemulsions. Carotenoids, PUFAs, and many other non-polar bioactive substances were demonstrated to have improved solubility and bioavailability when delivered via emulsion-based delivery systems [23,24,25]. The encapsulation process in this investigation followed the methods outlined by Gallardo et al. [26]. Specifically, gum Arabic was employed as the only wall material for encapsulating vegetable oil since the results of this study show a notable encapsulation efficiency of 90.5%.

The objective of the study was to explore the potential application of environmentally friendly extraction solvents, specifically edible vegetable oils, such as olive oil (OO), sunflower oil (SO), and flaxseed oil (FO), for the recovery of astaxanthin from shrimp by-products. Additionally, this study aimed to assess the encapsulation efficiency of the extracted astaxanthin. The obtained results could enhance the growing knowledge of eco-friendly extraction methods, particularly highlighting the potential significance of OO, which is a green solvent that has received limited attention in terms of extracting astaxanthin from shrimp by-products.

## 2. Results and Discussion

### 2.1. Fatty Acids Composition of Shrimp By-Product Total Lipids (TLs)

**Figure 2:**
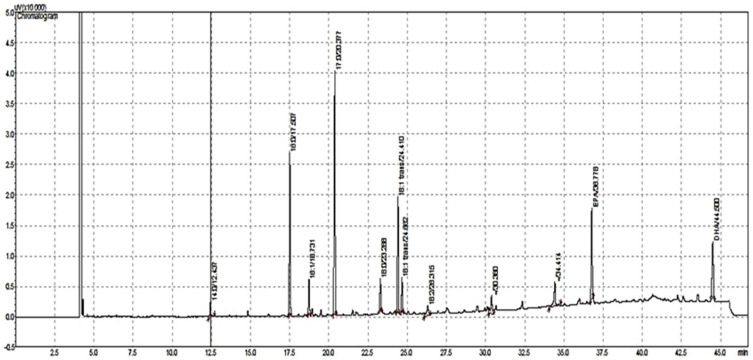
Representative chromatogram of shrimp by-product total lipids (TLs) using gas chromatography with a flame ionization detector (GC-FID).

The fatty acids composition of shrimp by-product total lipids (TLs) is presented in Table 1. A representative chromatogram of shrimp by-product TLs using gas chromatography with a flame ionization detector (GC-FID) is presented in Figure 2.

The distribution of shrimp by-product TLs was found to be saturated fatty acids (SFAs) (1.3622 g SFAs/kg shrimp by-products) > PUFAs (1.3485 g PUFAs/kg shrimp by-products) > monounsaturated fatty acids (MUFAs) (1.2428 g MUFAs/kg shrimp by-products) (Table 1).

The SFAs of shrimp by-product TLs were mainly comprised of palmitic acid (16:0) (1.0362 g 16:0/kg shrimp by-products) and stearic acid (18:0) (0.2134 g 18:0/kg shrimp by-products), while MUFAs exhibited elevated amounts of oleic acid (18:1 cis ω-9) (0.7983 g 18:1 cis ω-9/kg shrimp by-products) (Table 1). These findings are in agreement with the existing literature since shrimp oil derived from processed by-products contained a substantial quantity of C16:0 (15.73% (nmol/nmol)) and C18:0 (2.42% (nmol/nmol)), while the MUFAs contained mainly C18:1 cis ω-9 (21.33% (nmol/nmol)) [27]. Likewise, the oil obtained from by-products of deep-sea shrimp was found to encompass a notable concentration of SFAs. Specifically, palmitic acid (C16:0) constituted a substantial portion at 27.27 ± 0.68%, while among the MUFAs, oleic acid (C18:1) emerged as the predominant component with the highest prevalence, accounting for 15.81 ± 0.62% [28].

Within the group of PUFAs, shrimp by-product TLs exhibited high ω-3 PUFA content, with eicosapentaenoic acid (EPA, 20:5 ω-3) being the most prevalent at 0.6768 g EPA per kg of shrimp by-products, followed by docosahexaenoic acid (DHA, 22:6 ω-3) at 0.5418 g DHA per kg of shrimp by-products, whereas there was a small amount of ω-6 PUFAs, with linoleic acid (C18:2 ω-6) being the most abundant (0.1300 g C18:2 ω-6/kg shrimp by-products) (Table 1). Similar data were obtained from other researchers, as shrimp oil derived from processed by-products exhibited a high concentration of ω-3 PUFAs, with 21% EPA (C20:5 ω-3) and 13.89% DHA (C22:6 ω-3), and a small content of ω-6 PUFAs, with 1.96% C18:2 ω-6 [27].

Moreover, Sánchez-Camargo et al. [27] observed increased amounts of EPA and DHA in lipids extracted from red-spotted shrimp residue when using ethanol as a co-solvent [29]. More specifically, the researchers observed an improved extraction efficacy of EPA (from 5.91% to 11.48%) and DHA (from 4.29% to 12.24%) when using 15% ethanol as a co-solvent [29].

Similarly, Aneesh et al. [28] identified DHA (22:6, n-3) as a significant PUFA that was predominant in the extracted oil from shrimp in substantial proportions.

### 2.2. Radical Scavenging Activity of Shrimp By-Product TLs

The evaluation of shrimp by-product TLs’ radical scavenging activity using 2,2′-azinobis-(3-ethylbenzothiazoline-6-sulphonate) radical (ABTS^·+^) scavenging, 1,1-diphenyl-2-picrylhydrazyl (DPPH^·^) radical scavenging, ferric reducing antioxidant power (FRAP), and cupric ions (Cu^2+^) reducing power (Cuprac) assays is presented in Table 2.

The ABTS assay was found to yield the highest (ANOVA and Dunnett test, *p* < 0.05) antioxidant capacity of 81.2 ± 0.77 nmol trolox/mg of shrimp by-product TLs, followed by the CUPRAC, FRAP, and DPPH assays, with capacities equal to 37.0 ± 1.58, 1.81 ± 0.13, and 0.39 ± 0.01 nmol trolox/mg of shrimp by-product TLs, respectively. These results highlight the diverse antioxidant properties of the sample in different chemical environments, along with the ability to scavenge free radicals and exhibit antioxidant properties. These antioxidant properties of shrimp by-product TLs could be attributed to the carotenoid pigment content, especially astaxanthin, which is a bioactive compound with potent antioxidant activity and more effective radical scavenging activity than vitamin C and vitamin E [30,31,32].

### 2.3. Impact of Different Parameters on the Shrimp By-Product Astaxanthin UAE Efficiency

Astaxanthin UAE was performed at room temperature. The study focused on several crucial parameters, including the ratio of the shrimp by-product to vegetable oil, the duration of extraction, the amplitude of the extraction process, and the utilization of three different vegetable oils (OO, SO, and FO) as solvents for extraction. The values of the components under investigation, as well as the results gained from the trials conducted, are presented in Table 3, Table 4, Table 5 and Table 6.

#### 2.3.1. Impact of By-Product–Vegetable Oil Ratio on the Astaxanthin UAE Efficiency

Table 3 shows the impact of the by-product–SO ratio on the astaxanthin UAE yield. The experiment involved a systematic variation in the shrimp by-product–vegetable oil ratio, ranging from 1:10 to 1:80. Additionally, the extraction process lasted for 30 min, with a consistent amplitude of 100%.

The highest yield (67.3 ± 2.81 μg astaxanthin/g by-products) (ANOVA and Dunnett test, *p* < 0.05) was obtained at a ratio of 1:60, whereas the lowest yield (44.7 ± 1.33 μg astaxanthin/g by-products) was observed at a ratio of 1:10 (Table 3). Additionally, the astaxanthin yields obtained at 1:10, 1:20, 1:40, and 1:80 by-product–vegetable oil ratios were found to exhibit no significant differences (Table 3).

These results suggest that a rather balanced ratio of by-product–vegetable oil contributed to a higher astaxanthin extraction yield. These observations align with the findings in the existing literature. According to Pok et al. [17], they observed an astaxanthin extraction yield from crab of 50 ± 5 μg/g of crab, which is comparatively lower than the one obtained in the current study, by employing soybean oil as a solvent, along with a rather excessive oil-to-crab ratio of 140 mL/g. Furthermore, in the study of Sachindra and Mahendrakar [15], the yield of extracted carotenoids from shrimp waste using SO as a solvent under optimized conditions, including a rather low oil-to-waste ratio of 2:1, was found to be less than half (27.56 μg/g of shrimp waste) in comparison with the one obtained in the present study at a ratio of 1:60.

#### 2.3.2. Impact of Extraction Time on the Astaxanthin UAE Efficiency

##### Table 4 Presents the Impact of Extraction Duration on Astaxanthin UAE Efficiency

The extraction process was conducted with an amplitude of 100% and a shrimp by-product–SO ratio of 1:60.

Longer extraction times generally resulted in higher astaxanthin extraction yields. The maximum yield (155 ± 3.83 μg astaxanthin/g by-products) (ANOVA and Dunnett test, *p* < 0.05) was achieved at 210 min, followed by 120 and 240 min with astaxanthin yields of 74.5 ± 5.26 μg/g and 74.0 ± 3.29 μg/g, respectively (Table 4). However, the yield significantly dropped to 74.0 ± 3.29 μg astaxanthin/g by-products at 60 min (Table 4).

Such results indicate that sufficient extraction time is necessary for efficient astaxanthin recovery. The literature supports the fact that sufficient extraction time is indispensable for achieving efficient astaxanthin recovery, as evidenced by successful astaxanthin extraction from shrimp waste using SO as a solvent under optimized conditions, which involved a heating time of 150 min to attain the maximum extraction efficiency [15]. Similarly, the utilization of soybean oil for astaxanthin extraction from crab oil, following optimized conditions that involved a 170 min duration [17], and from shrimp residues through 160 min extraction process at room temperature [1] yielded the maximum astaxanthin content.

#### 2.3.3. Impact of Amplitude on Astaxanthin UAE Efficiency

Table 5 displays the impact of amplitude on the astaxanthin UAE yield. The extraction procedure was carried out over a duration of 210 min and with the shrimp by-product–SO ratio set to 1:60.

An amplitude of 60% led to the highest yield of 133.0 ± 4.57 μg astaxanthin/g by-products (ANOVA and Dunnett test, *p* < 0.05), while a 40% amplitude resulted in a yield of 104.9 ± 4.2 μg astaxanthin/g by-products (Table 5). However, using a 100% amplitude significantly decreased the astaxanthin yield to 67.3 ± 2.81 μg astaxanthin/g by-products (Table 5). These findings suggest that an amplitude of 60% holds significant implications for an effective UAE process, underlining the fact that moderate amplitudes are more suitable for astaxanthin extraction compared with lower or higher amplitudes.

Sharayei et al. [33] used an ultrasonic amplitude of 23.6% for astaxanthin extraction, and Prayitno et al. [16], who utilized 40% amplitude for extracting astaxanthin from cincalok, further complement and support the findings of the current study. According to the obtained results, an amplitude of 60% seems to strike a balance, efficiently breaking down the shrimp by-products to release astaxanthin while avoiding potential adverse effects from excessive energy input. Lower amplitudes might not provide sufficient energy to effectively disrupt the cell structures and release astaxanthin, resulting in lower yields. On the other hand, higher amplitudes might lead to excessive energy input, causing potential degradation of astaxanthin molecules and leading to reduced yield. Moreover, the amplitude’s influence on astaxanthin extraction can be attributed to the physical properties of the sample and the solvent’s interaction with ultrasonic waves.

#### 2.3.4. Impact of Vegetable Oil Solvent on the Astaxanthin UAE Efficiency

Table 6 compares the astaxanthin UAE yields using different vegetable oils as extraction solvents. The extraction process was conducted for 210 min, utilizing a shrimp by-product–vegetable oil ratio established at 1:60, along with an amplitude of 60%.

OO exhibited the highest efficiency, resulting in a yield of 235 ± 4.07 μg astaxanthin/g by-products (ANOVA and Dunnett test, *p* < 0.05) (Table 6). FO followed with a yield of 117 ± 4.45 μg astaxanthin/g by-products (Table 6). In contrast, SO resulted in a significantly lower yield of 90.2 ± 5.87 μg astaxanthin/g by-products (Table 6). This implies that the choice of solvent significantly impacted the astaxanthin extraction efficiency.

According to the current study, OO stood out as a green and sustainable extraction solvent, exhibiting remarkable efficacy in improving the shrimp by-product astaxanthin UAE yield. The obtained results demonstrated that using OO as the extraction solvent resulted in the highest astaxanthin UAE efficiency compared with SO and FO. The green nature of OO makes it an environmentally friendly choice, aligning with the growing demand for sustainable extraction processes. Its high lipid content and unique composition likely facilitate enhanced astaxanthin solubility, leading to a more efficient extraction. As a result, incorporating OO as the extraction solvent holds great promise in maximizing astaxanthin yields while minimizing the environmental impact, making it a preferred choice for future applications in the food and nutraceutical industries.

In summary, the acquired findings show that the extraction conditions significantly affected the yield of shrimp by-product astaxanthin. Based on the results provided, a by-product–vegetable oil ratio of 1:60, extraction time of 210 min, 60% amplitude, and the utilization of OO as the extracting medium, a significant astaxanthin yield of 235 ± 4.07 μg astaxanthin/g by-products was produced.

### 2.4. UAE Yield of the Shrimp By-Product Astaxanthin Using Three Different Plant Oils—Accelerated Stability Study

Three different plant oils, namely, OO, SO, and FO, were examined as possible solvents to maximize the shrimp by-product astaxanthin UAE yield. The extraction procedure’s outcomes are displayed in Table 7.

The astaxanthin extraction efficiency depends on the plant oil used as the extraction solvent. The astaxanthin yield was found to be significantly increased (ANOVA and Duncan test, *p* < 0.05) when using OO as the extraction solvent during UAE (94.4 ± 1.01 μg astaxanthin/g by-products) compared with SO (70.1 ± 1.39 μg astaxanthin/g by-products) and FO (72.2 ± 1.52 μg astaxanthin/g by-products) on day 0. The same trend was observed during the accelerated stability test on subsequent days, indicating the sustained efficacy of OO as an extraction medium (Table 7).

The increased astaxanthin extraction yield observed when using OO may be attributed to its high oleic acid content, along with the endogenous amphiphilic content [34].

Moreover, on day 1, the astaxanthin extraction efficiency was found to be significantly elevated when using FO (66.1 ± 1.26 μg astaxanthin/g by-products) as the extraction solvent compared with SO (55.7 ± 1.0 μg astaxanthin/g by-products), while during the second, third, and fourth days of the accelerated study, FO and SO were found to exhibit similar trends, with decreasing astaxanthin content over time. The elevated astaxanthin extraction yield observed using FO on day 1 could be attributed to the fact that FO is characterized by decreased viscosity compared with SO [35]. Low solvent viscosity is usually related to higher solvent penetration into the matrix by enabling ultrasonic wave distribution and cavitation bubble formation, resulting in improved extraction efficiency [36].

Astaxanthin recovery using all three extraction solvents (OO, SO, and FO) was found to gradually decrease during the acceleration stability test, though it was maintained at a considerably high percentage when using OO on day six (80.3 ± 4.3%) (Table 7).

The level of PUFAs in triglycerides present in vegetable oils [34], along with the fact that astaxanthin, a lipophilic pigment, is characterized by increased solubility in oil [12] make vegetable oils, such as OO, FO, and SO potential, alternative green extraction solvents for recovering natural pigments, replacing organic solvents, and minimizing environmental impacts [18]. Another advantage of vegetable oils is that they can act as an oxygen barrier that protects the extracted astaxanthin from oxidation and therefore prolongs the shelf-life [15].

The usage of OO as an extraction solvent for astaxanthin from shrimp by-products in UAE highlights for the first time the potential use of OO as a green and effective solvent for astaxanthin UAE from shrimp by-products, offering promising prospects for sustainable and efficient extraction processes in the food industries. The utilization of OO not only enhances astaxanthin recovery but also adds value to shrimp by-product utilization, contributing to the circular economy and reducing the environmental impact.

OO has also been used to recover astaxanthin from encysted *Haematococcus* culture through the direct extraction process. The evaluation of OO, soybean oil, corn oil, and grapeseed oil for the recovery of astaxanthin from encysted *Haematococcus* culture indicates that OO exerts a higher extraction yield than other plant oils [37].

Sachindra and Mahendrakar [15] used different vegetable oils, such as SO, soya oil, and coconut oil, for the recovery of astaxanthin from shrimp waste. SO was found to exert the highest astaxanthin yield (26.3 ± 2.31 μg/g waste) [15], a result which is approximately 3 times lower compared with the respective result of the present study; the astaxanthin yield using SO during UAE on day 0 was 70.1 ± 1.39 μg astaxanthin/g shrimp by-product (Table 7). Similarly, recent research involving the utilization of SO for extracting astaxanthin from crab exhibited that under optimized conditions, including a ratio of 60 mL/g for oil to crab, along with an extraction time of 161 min, yielded 31 ± 3 μg astaxanthin/g of crab dry matter [17], which is more than two times lower than the corresponding outcome of the present investigation; the astaxanthin yield using SO during UAE on day 0 was 70.1 ± 1.39 μg astaxanthin/g shrimp by-product (Table 7). This large difference regarding astaxanthin extraction efficiency may be attributed to the implementation of UAE, by which ultrasonic waves create a cavitation effect, resulting in cell disruption, facilitating solvent penetration into the matrix, and therefore, improving the extraction efficiency.

Likewise, previously reported data revealed that FO and SO exhibited astaxanthin recovery from shrimp wastes up to 4.89 μg astaxanthin/g waste and 3.70 μg astaxanthin/g waste, respectively [38], results which are significantly lower compared with the respective result of the present study; the astaxanthin yield was 72.2 ± 1.52 μg astaxanthin/g shrimp by-product when using FO and 70.1 ± 1.39 μg astaxanthin/g shrimp by-product when using SO during UAE on day 0 (Table 7). Such variation was due to UAE, underlying the effectiveness of this extraction method.

### 2.5. Radical Scavenging Activity of the Shrimp By-Product Astaxanthin Obtained Using UAE in Three Different Plant Oils as Extraction Solvents

The antioxidant capacity of the astaxanthin obtained from the UAE of shrimp by-products using three different plant oils as extraction solvents over six days was evaluated using ABTS radical cation scavenging activity and was expressed as the concentration that scavenged 50% of the ABTS radical cation (IC_50_). Table 8 summarizes the findings as determined by the ABTS assay.

According to Table 8, the astaxanthin obtained using both OO and SO was found to exhibit low IC_50_ values on day 0, with 2.2 ± 0.03 and 1.60 ± 0.02 μg astaxanthin, respectively, while astaxanthin recovered using FO was found to exert an elevated IC_50_ value on day 0 (13.4 ± 0.27 μg astaxanthin) (Table 8).

At this point, it should be mentioned that the lower the IC_50_ value, the stronger the antioxidant capacity a sample exerts. Therefore, astaxanthin extracted using SO, as a green extraction solvent, during UAE was found to exert more potent radical scavenging activity (1.60 ± 0.02 μg astaxanthin) compared with astaxanthin extracted using OO as a green extraction solvent (2.2 ± 0.03 μg astaxanthin) (Table 8). These findings may appear counterintuitive, as OO is known for its high antioxidant content and health-promoting properties. However, it should be noted that the radical scavenging ability of astaxanthin can be influenced by various factors, including its interaction with other components in the extraction solvent. Therefore, the enhanced radical scavenging capacity of astaxanthin extracted using SO could be attributed to the increased PUFA content of SO, mainly consisting of linoleic acid (18:2, ω-6), compared with OO [39] in combination with the synergistic interaction between such PUFAs and astaxanthin in terms of radical scavenging activity [12].

The results obtained from the accelerated stability test revealed noteworthy findings regarding the antioxidant activity of astaxanthin extracted using different green solvents. Throughout the experiment, astaxanthin obtained using SO as extraction solvent demonstrated consistently higher radical scavenging capacity on days 0, 1, 3, 4, and 5 compared with astaxanthin recovered using OO (Table 8), indicating that SO was found to be more effective in preserving the potent antioxidant properties of astaxanthin.

However, it is worth noting that on days 2 and 6, the antioxidant activity of astaxanthin extracted with OO was temporarily found to exert more potent radical scavenging activity than that of the SO-extracted astaxanthin. These results suggest that while SO was found to be a more powerful solvent for preserving astaxanthin’s radical scavenging capacity overall, there may be unique properties of OO that contribute to temporary enhancements in antioxidant activity at specific time points during the accelerated stability test.

The enhanced activity of SO can also be attributed to the increased vitamin E content present in SO compared with OO [40]. Vitamin E plays a crucial role in exerting a protective effect against astaxanthin auto-oxidation, acting as a potent antioxidant within biological systems. Astaxanthin, which is a carotenoid pigment with remarkable antioxidant properties that are mainly attributed to the conjugated double bonds that act as electron donors to stabilize free radicals [12], can undergo auto-oxidation when exposed to various environmental stressors, such as oxygen, leading to the formation of various auto-oxidation products [41].

However, when vitamin E is present, it serves as a sacrificial antioxidant, donating electrons to neutralize these free radicals and preventing them from attacking other cellular components. This synergistic interaction between vitamin E and astaxanthin ensures a reinforced defense against oxidative damage, making them valuable components of a protective diet [42].

Overall, these findings indicate that although SO exhibits superior efficacy as a solvent for preserving astaxanthin’s radical scavenging capability on the whole, there might be distinct characteristics of OO that contribute to improvements in antioxidant activity at particular time intervals during the accelerated stability assessment.

### 2.6. Astaxanthin Encapsulation Efficiency—Accelerated Stability Study

The diameter of the particles was determined to be 15.8 ± 3.8 μm and the moisture of the encapsulated material was 2.5 ± 0.4%.

The encapsulation efficiency (% E.E) indicates the percentage of shrimp by-product astaxanthin generated using the UAE method and OO as the extraction solvent that was successfully encapsulated, whereas the % astaxanthin recovery represents the percentage of astaxanthin recovered after the encapsulation process. Both the encapsulation efficiency (% E.E) and astaxanthin recovery provide valuable insights into the stability and effectiveness of the encapsulation process over the course of the study. On day 0, the astaxanthin encapsulation efficiency was notably high at 66.6 ± 2.7% (ANOVA and Dunnett test, *p* < 0.05) (Table 9), indicating that a substantial amount of astaxanthin was successfully encapsulated within the delivery system (mixture of gum Arabic and soy lecithin). The astaxanthin recovery on day 1 was found to be impressively high at 94.4 ± 4.6% (ANOVA and Dunnett test, *p* < 0.05) (Table 9), indicating that the majority of the encapsulated astaxanthin was successfully recovered after the encapsulation process.

Similar outcomes on the encapsulation performance were reported by Xie et al. [41], who encapsulated astaxanthin-enriched camelina oil extract when emulsified using a mixture of egg albumin (EA) and gum Arabic (GA), both with and without tannic acid cross-linking. The entrapment efficiency of astaxanthin in the stabilized emulsions using EA/GA was observed to be around 70%. However, it is noteworthy that approximately 30% of astaxanthin was lost during the emulsion preparation process. The authors attributed this loss to oxidation that likely occurred during the two steps of homogenization involved in the emulsification process. The homogenization steps might have generated free radicals, leading to a 20% loss of astaxanthin [43]. Gomez-Estaca et al. [42] reported the encapsulation of shrimp waste astaxanthin in gelatin and cashew gum coacervates, achieving an encapsulation efficiency of 59.9% [44].

Moreover, the acquired results revealed that even on the fifth day during the accelerated stability study, both the % E.E of astaxanthin and the % astaxanthin recovery exerted elevated values (Table 9), signifying the continued effectiveness and stability of the encapsulation process. However, it is essential to acknowledge the overall gradual decline in both parameters over time, indicating the potential need for further optimization in the formulation and storage conditions to ensure prolonged stability and enhanced encapsulation efficiency.

## 3. Materials and Methods

### 3.1. Materials

All reagents and solvents were of analytical grade and purchased from Merck (Darmstadt, Germany). Gum Arabic (GA) was obtained from Sosa Ingredients (Moià, Catalonia, Spain). OO and SO were purchased from a local supermarket in Myrina, Lemnos, while FO was purchased from a local bioproduct market in Athens, Greece.

### 3.2. Sample Collection and Preparation

Shrimp (*Penaeus longirostris*) by-products (head, shell, and tail) were obtained from a local fish market (Myrina, Lemnos), washed with distilled water, and then dried using a food dryer (24 h at 70 °C) as previously reported [45]. Dry shrimp by-product samples were processed for one minute in a laboratory grinder IKA A 10 basic (IKA Works, Wilmington, DE, USA) in order to produce a sample of fine powder.

### 3.3. Isolation of Shrimp By-Product Total Lipids (TLs)

The extraction of total lipids (TLs) was carried out following the method of Bligh-Dyer [46]. Briefly, a known amount of shrimp by-product fine powder (2 g) was added to an appropriate amount of chloroform/methanol/water 1:2:0.8 *v*/*v*/*v* solution. The mixture was well agitated. Chloroform and water were added to the mixture in the separatory funnel to achieve phase separation, resulting in a final chloroform/methanol/water ratio of 1/1/0.9 *v*/*v*/*v*. The chloroform phase (lower phase) was evaporated to dryness using nitrogen gas in order to obtain the TLs. The lipids were weighed and redissolved in 5 mL chloroform:methanol 1:1 *v*/*v* and stored at −20 °C until used for the fatty acid analysis.

### 3.4. Fatty Acid Analysis of Shrimp By-Product TLs

Fatty acid methyl esters of shrimp by-product TLs were prepared using a solution 0.5 M KOH in CH_3_OH (KOH–CH_3_OH method), as previously described by Nasopoulou et al. [47]. The formed FA methyl esters (FAMEs) were extracted with hexane and analyzed using the internal standard method as previously stated [48] with slight modifications.

The fatty acid analysis was performed using a Shimadzu CLASS-VP (GC-17A) (Kyoto, Japan) gas chromatograph equipped with a split/splitless injector and flame ionization detector.

Separation of the fatty acid methyl esters was achieved on an Agilent J&W DB-23 fused silica capillary column (60 m × 0.251 mm i.d., 0.25 μm; Agilent, Santa Clara, CA, USA). The temperature program used started at 150 °C and remained there for 5 min, and then the temperature increased at a rate of 2 °C/min until 215 °C, where it remained for 8 min.

### 3.5. Radical Scavenging Activity

2,2′-azinobis-(3-ethylbenzothiazoline-6-sulphonate) radical (ABTS^·+^) scavenging, 1,1-diphenyl-2-picrylhydrazyl (DPPH^·^) radical scavenging, ferric reducing antioxidant power (FRAP), and cupric ion (Cu^2+^) reducing power (Cuprac) assays were conducted by utilizing the technique described by Michalaki et al. [49]. In each measurement, a blank sample that was the corresponding amount of solvent (OO, SO, or FO) was used. Each sample was examined in triplicate. The results are expressed as Trolox equivalents in nmol per mg of shrimp by-product total lipids for all antioxidant tests. In addition, the antioxidant activity of astaxanthin obtained using UAE in different green extraction solvents is expressed in micrograms of astaxanthin. 

### 3.6. Study of the Impact of the By-Product–Vegetable Oil Ratio, Extraction Time, Amplitude, and Vegetable Oil Solvents on Shrimp By-Products Astaxanthin UAE Efficiency

The UAE of shrimp by-product astaxanthin was carried out at room temperature via a series of experiments. The study focused on many crucial characteristics, namely, the ratio of shrimp by-product to vegetable oil, extraction time, amplitude, and the choice of different vegetable oils as solvents for extraction. The shrimp by-product–vegetable oil ratio was systematically varied from 1:10 to 1:80, while the extraction time was examined across a range of 60 min to 240 min. The amplitude of the extraction process was assessed at three different levels: 40%, 60%, and 100%. To comprehensively explore the impact of different extraction solvents, three distinct vegetable oils, namely, OO, SO, and FO, were employed. The objective of these tests was a high level of astaxanthin extraction while also ensuring an environmentally sustainable and energy-efficient extraction process.

### 3.7. UAE of the Shrimp By-Product Astaxanthin in Different Vegetable Oils

The UAE was carried out using an ultrasonic processor VCX-750 equipped with a sealed converter (Sonics & Materials, Inc., Newtown, CT, USA) with an ultrasound power of 750 W and a frequency of 37 KHz.

Vegetable oils OO, SO, and FO were used as alternative extraction solvents. The powder of the shrimp by-products was accurately weighed, placed in capped tubes in a ratio of 1:60 (g/mL of oil), and homogenized with an appropriate amount of solvents. After wetting the material, the tubes with the suspensions were immersed into a cup horn with a 1.000 mL maximum volume in the ultrasonic device and irradiated for the predetermined extraction time. After the ultrasonic extraction, the sample was centrifuged at 20,000× *g* for 20 min and the supernatant was collected. The extracts were measured using a Lambda 25 spectrophotometer (Perkin Elmer, Norwalk, CT, USA).

### 3.8. Yield of Carotenoids in Different Vegetable Oils

To determine the carotenoid yield, the total carotenoid content, specifically astaxanthin, in each vegetable oil was measured spectrophotometrically [50]. A blank measurement was taken for each vegetable oil to account for the background absorption. The carotenoid yield was then calculated using the Lambert–Beer equation, incorporating the respective extinction coefficients for each vegetable oil. The extinction coefficients used were 50,988 for OO, 50,429 for SO, and 50,477 for FO. These extinction coefficients were obtained through the construction of astaxanthin standard curves for each vegetable oil, allowing for accurate quantification of the astaxanthin content in the samples.

### 3.9. Encapsulation Process

#### 3.9.1. Preparation of Microcapsules

Oil-in-water (O/W) emulsions were prepared using gum Arabic as the wall material, while soy lecithin was used as an emulsifier. The encapsulating agent (48 g) was dissolved in deionized water (112 mL) with constant stirring at room temperature (25 °C) for 2 h. The solution was left at 8 °C overnight to ensure total hydration. Subsequently, 12 mL of OO with astaxanthin extract and 1.7 g of soy lecithin were added to the wall material. Then, the mixture was homogenized at 16,000× *g* for 8 min at room temperature using a homogenizer (Unidrive X1000D Homogenizer, CAT Scientific, Paso Robles, CA, USA) with a 17 mm diameter shaft (Ingenieurbuero CAT, M. Zipper GmbH, Ballrechten-Dottingen, Germany). The emulsions were then transferred to a deep freezer (−80 °C) for 24 h, and freeze drying was carried out utilizing a BK-FD10PT freeze dryer (Biobase Biodustry Co., Ltd., Jinan, China) for 48 h. To produce a fine encapsulated powder, the dried material was shattered in an IKA A 10 basic lab grinder (IKA Works, Wilmington, DE, USA). The moisture content of the samples was calculated from the weight loss after heating 30 g of the sample in triplicate in a drying oven (Binder GmbH, ED 115, Tuttlingen, Germany) at 105 °C for 24 h.

#### 3.9.2. Encapsulation Efficiency (E.E.)

Determination of the encapsulation efficiency (% E.E.) of shrimp by-product astaxanthin in microcapsules involved acid hydrolysis and solvent extraction, as described by Gallardo et al. [26]. Briefly, one gram of powder was carefully placed in a glass tube and 10 mL of 9.5 mol/L hydrochloric acid was slowly added. The tubes were put in a 70 °C water bath for 30 min. After cooling to 25 °C, 25 mL of ethyl and petroleum ether were added to the tubes. After that, the tubes were vigorously shaken for one minute and then the ether solution supernatant was filtered. The remaining aqueous phase was extracted twice with 15 mL of ethyl and petroleum ether. The solvent was then evaporated using a rotary evaporator (LabTech, Inc. Hapkinton, MA, USA). The oil was then used to quantify astaxanthin (see Section 3.8). For carotenoids in non-encapsulated oil, 4 g of microcapsule powder was drip-washed in 75 milliliters of ethyl ether for 15 min at 25 °C. Three ethyl ether rinses of the powder residue on a Whatman No. 1 filter paper filtered the suspension. The solvent was dried and rotary evaporated to obtain the surface oil mass, where the non-encapsulated astaxanthin was quantified (see Section 3.8).

The encapsulation efficiency (EE%) was calculated using the following equation:

$$\mathrm{EE}\%=\left(\frac{\mathrm{TAsx}-\mathrm{NEAsx}}{\mathrm{TAsx}}\right)\times 100$$

where TAsx is the total astaxanthin content and NEAsx is the non-encapsulated astaxanthin content.

#### 3.9.3. Particle Size Analysis and Moisture Content

Particles of the sample were observed with a BA210E microscope and Moticam 3.0 MP at 40× objective magnification (Motic Spain SL, Barcelona, Spain). The sample was placed on glass slides and covered with a coverslip. The particle analysis was performed using Image J version 1.53t (National Institutes of Health, Bethesda, MD, USA). The counting area of each optical image was randomly selected, and the diameters of 300 particles were measured. The results were expressed as the mean value ± standard deviation.

### 3.10. Accelerated Stability Study

An accelerated stability study aims to examine the vulnerability to oxidation, which involves subjecting the samples to an accelerated oxidation test under controlled and standardized conditions. The samples of extracted astaxanthin using three vegetable oils, the three vegetable oils as blank samples, the encapsulated astaxanthin extracted using OO, and the encapsulated OO as a blank were carefully sealed and placed in an oven set at 65 °C for a total duration of six days. At regular intervals of 24 h, samples were extracted for analysis as described in Section 3.9.2. This accelerated oxidation test was established in prior research to simulate the effects of long-term storage at room temperature. Specifically, one day under accelerated test conditions is considered equivalent to one month of storage at room temperature [51].

### 3.11. Statistical Analysis

Statistical analysis was conducted using the SPSS^®®^ computer program (SPSS Statistical Software, Inc., version 29, Chicago, IL, USA). A one-way analysis of variance (ANOVA) with Dunnett’s *t*-test was used for one independent variable and a two-way analysis of variance (ANOVA) with Duncan’s post hoc test was employed to assess the differences between two independent variables in order to compare the differences between the means of different groups, e.g., different vegetable oils and different days. The level of significance was set at *p* < 0.05, indicating that any observed differences with a probability of occurrence of less than 5% were considered statistically significant.

## 4. Conclusions

A balanced by-product–vegetable oil ratio, longer extraction time, and moderate amplitude were found to enhance the astaxanthin extraction efficiency. Furthermore, the choice of vegetable oil solvent significantly impacted the yield, with OO exhibiting the highest astaxanthin extraction efficiency. OO utilization as a green and sustainable extraction solvent for astaxanthin derived from shrimp by-products in UAE represents for the first time a noteworthy and innovative approach for various applications in the food and nutraceutical industries, where astaxanthin’s potent antioxidant and health-promoting properties can be harnessed to create functional and value-added products.

Astaxanthin, which was obtained via UAE using SO as a solvent, displayed notable efficacy in scavenging radicals. Although OO is known for its abundant antioxidants, the utilization of SO in the UAE process seemed to amplify astaxanthin’s capacity for radical scavenging, likely owing to its content of PUFAs and alpha-tocopherol. Nevertheless, additional investigation is required to gain a comprehensive understanding of the factors that contributed to these divergent outcomes.

Moreover, a delivery system comprising a mixture of gum Arabic and soy lecithin demonstrated successful encapsulation of astaxanthin extracted using OO, along with effective astaxanthin recovery within the accelerated stability test, offering promising prospects for utilizing this delivery system in food and nutraceuticals industries, and therefore, to enhance the bioavailability and potential health benefits of astaxanthin.

Overall, this study contributes to the growing knowledge of green extraction methods, underlining the utilization of OO as a green extraction solvent, offering valuable insights for the development of antioxidant-enriched products, the promotion of sustainable and eco-friendly extraction and encapsulation practices, and contributing at the same time to the growing efforts in sustainable waste utilization.

## Figures and Tables

**Table 1:** Fatty acids composition of shrimp by-product TLs (means ± SD, n = 6) expressed in g of fatty acids per kg of shrimp by-products.

Fatty Acid	g/kg of Shrimp By-Products
Myristic acid 14:0	0.1126 ± 0.0066
Palmitic acid 16:0	1.0362 ± 0.0391
Stearic acid 18:0	0.2134 ± 0.0119
Palmitoleic acid 16:1 (ω-7)	0.2195 ± 0.0085
Oleic acid 18:1 *cis* (ω-9)	0.7983 ± 0.0312
Elaidic acid 18:1 *trans* (ω-9)	0.2250 ± 0.0125
Linoleic acid 18:2 (ω-6)	0.1300 ± 0.0074
Eicosapentaenoic acid 20:5 (ω-3)	0.6768 ± 0.0364
Docosahexaenoic acid 22:6 (ω-3)	0.5418 ± 0.0320
Total SFAs	1.3622 ± 0.0809
Total MUFAs	1.2428 ± 0.0727
Total ω-3 PUFAs	1.2186 ± 0.0481
Total ω-6 PUFAs	0.1300 ± 0.0520
ω-3/ω-6	9.3718 ± 0.0006

SFAs: saturated fatty acids; MUFAs: monounsaturated fatty acids; PUFAs: polyunsaturated fatty acids.

**Table 2:** Antioxidant activities of shrimp by-product TLs (mean ± SD, n = 3) expressed in nmol of Trolox per mg of TL.

Assay	nmol Trolox/mg TL
ABTS	81.2 ± 0.77 ^a^
CUPRAC	37.0 ± 1.58 ^b^
FRAP	1.81 ± 0.13 ^c^
DPPH	0.39 ± 0.01 ^c^

^a,b,c^: different letters indicate significantly different values among different assays (*p* < 0.05), according to a one-way ANOVA.

**Table 3:** Astaxanthin UAE yields at different by-product–SO ratios (*w*/*v*) (mean ± SD, n = 3) expressed in μg astaxanthin/g by-products.

By-Product–SO Ratio (*w*/*v*)	(μg Astaxanthin/g By-Products)
1:10	44.7 ± 1.33 ^a^
1:20	45.8 ± 1.68 ^a^
1:40	49.0 ± 2.43 ^a^
1:60	67.3 ± 2.81 ^b^
1:80	51.2 ± 3.86 ^a^

SO: sunflower oil; ^a,b^: different letters indicate significantly different values between different by-product–SO ratios (*p* < 0.05) according to a one-way ANOVA.

**Table 4:** Astaxanthin UAE yield at different extraction times (mean ± SD, n = 3) expressed in μg astaxanthin/g by-products.

Extraction Time (min)	(μg Astaxanthin/g By-Products)
60	52.7 ± 3.57 ^a^
120	74.5 ± 5.26 ^b^
210	155 ± 3.83 ^c^
240	74.0 ± 3.29 ^b^

^a,b,c^: different letters indicate significantly different values between different extraction times (*p* < 0.05) according to a one-way ANOVA.

**Table 5:** Astaxanthin UAE yields at different amplitudes (mean ± SD, n = 3) expressed in μg astaxanthin/g by-products.

Amplitude	(μg Astaxanthin/g By-Products)
40%	104.9 ± 4.2 ^a^
60%	133.0 ± 4.57 ^b^
100%	67.3 ± 2.81 ^c^

^a,b,c^: different letters indicate significantly different values between different amplitudes (*p* < 0.05) according to a one-way ANOVA.

**Table 6:** Astaxanthin UAE yield using different vegetable oils as extraction solvents (mean ± SD, n = 3) expressed in μg astaxanthin/g by-products.

Vegetable Oil	(μg Astaxanthin/g By-Products)
SO	90.2 ± 5.87 ^a^
FO	117 ± 4.45 ^b^
OO	235 ± 4.07 ^c^

SO: sunflower oil, FO: flaxseed oil, OO: olive oil; ^a,b,c:^ different letters indicate significantly different values between different vegetable oils (*p* < 0.05) according to a one-way ANOVA.

**Table 7:** Shrimp by-product astaxanthin UAE yield expressed in μg astaxanthin/g by-products and accelerated stability using three different plant oils as solvents (mean ± SD, n = 3).

	Sample	μg Astaxanthin/g By-Products	% Astaxanthin Recovery	% Astaxanthin Loss
**Day 0**	OO	94.4 ± 1.01 ^a^	n.d.	n.d.
	SO	70.1 ± 1.39 ^b^	n.d.	n.d.
	FO	72.2 ± 1.52 ^c^	n.d.	n.d.
**Day 1**	OO	92.3 ± 1.01 ^a^	99.4 ± 4.6 ^a^	0.60 ± 0.00 ^a^
	SO	55.7 ± 1.0 ^d^	79.5 ± 3.7 ^b^	20.5 ± 1.02 ^b^
	FO	66.1 ± 1.26 ^e^	91.5 ± 3.7 ^c^	8.5 ± 0.39 ^c^
**Day 2**	OO	88.5 ± 1.13 ^f^	96.4 ± 4.2 ^a,d^	3.60 ± 0.18 ^d^
	SO	53.6 ± 0.96 ^d^	76.5 ± 3.9 ^d,e,f^	23.5 ± 0.94 ^e^
	FO	55.6 ± 1.0 ^d^	77.0 ± 3.3 ^e^	23.0 ± 1.17 ^e^
**Day 3**	OO	84.3 ± 0.93 ^g^	91.8 ± 3.4 ^a,d^	8.20 ± 0.39 ^f^
	SO	48.7 ± 1.09 ^h^	69.5 ± 3.6 ^f,g,h^	30.5 ± 1.32 ^g^
	FO	48.7 ± 0.96 ^h^	67.4 ± 2.8 ^g,i^	32.6 ± 1.70 ^g^
**Day 4**	OO	80.6 ± 0.96 ^i^	88.0 ± 4.0 ^d,j^	12.0 ± 0.68 ^h^
	SO	45.3 ± 0.84 ^j^	64.6 ± 3.3 ^h,k,l^	35.4 ± 1.69 ^i^
	FO	45.9 ± 0.96 ^j^	63.6 ± 3.4 ^i,k^	36.4 ± 1.60 ^i^
**Day 5**	OO	75.7 ± 0.97 ^k^	84.1 ± 4.1 ^d,j^	15.9 ± 0.83 ^j^
	SO	42.5 ± 0.73 ^l^	60.6 ± 2.5 ^h,l^	39.4 ± 2.10 ^k^
	FO	n.d.	n.d.	n.d.
**Day 6**	OO	69.9 ± 1.06 ^m^	80.3 ± 4.3 ^j^	19.7 ± 0.92 ^l^
	SO	41.8 ± 0.60 ^l^	59.6 ± 2.8 ^l^	40.4 ± 2.11 ^k^
	FO	n.d.	n.d.	n.d.

OO: olive oil, SO: sunflower oil, FO: flaxseed oil, n.d.: not detectable. ^a–m:^ different letters within a column indicate significantly different values between different pairs (*p* < 0.05) according to a two-way ANOVA with the type of vegetable oil and days being the two independent variables.

**Table 8:** Radical scavenging activity of the shrimp by-product astaxanthin using three different plant oils as extraction solvents (as mean ± SD, n = 3) expressed as the concentration that scavenged 50% of the ABTS radical cation (IC_50_).

	IC_50_ (μg Astaxanthin)		
Day	OO	SO	FO
0	2.2 ± 0.03 ^a^	1.60 ± 0.02 ^f^	13.4 ± 0.27 ^k^
1	2.8 ± 0.19 ^b^	1.97 ± 0.08 ^g^	n.d.
2	2.5 ± 0.12 ^a,b^	3.87 ± 0.23 ^h^	n.d.
3	3.2 ± 0.11 ^c^	2.73 ± 0.11 ^i^	n.d.
4	4.4 ± 0.20 ^d^	2.99 ± 0.05 ^i^	n.d.
5	3.3 ± 0.16 ^c^	2.86 ± 0.09 ^i^	n.d.
6	3.7 ± 0.09 ^e^	10.1 ± 0.24 ^j^	n.d.

OO: olive oil, SO: sunflower oil, FO: flaxseed oil; IC_50_: concentration that scavenged 50% of the ABTS radical cation; for each measurement, a blank sample that was the corresponding amount of solvent (OO, SO, or FO) was used. n.d.: not detectable; ^a–k:^ different letters indicate significantly different values between different pairs (*p* < 0.05) according to a two-way ANOVA with the type of vegetable oil and different days being the two independent variables.

**Table 9:** Astaxanthin encapsulation efficiency (% E. E.) and accelerated stability of the encapsulated astaxanthin (as mean ± SD, n = 3).

	% E. E.	% Astaxanthin Recovery
Day 0	66.6 ± 2.7 ^a^	-
Day 1	62.9 ± 2.6 ^a,b^	94.4 ± 4.6 ^a^
Day 2	56.5 ± 2.8 ^b^	84.9 ± 4.3 ^a,b^
Day 3	52.6 ± 3.1 ^b,c,e^	78.9 ± 3.9 ^b,c^
Day 4	48.0 ± 2.3 ^e,f^	72.1 ± 2.9 ^c,d^
Day 5	42.0 ± 1.8 ^f^	63.0 ± 2.4 ^d^
Day 6	28.9 ± 1.6 ^g^	43.4 ± 2.2 ^e^

% E.E.: encapsulation efficiency. ^a–g:^ different letters within a column indicate significantly different values between different days (*p* < 0.05) according to a one-way ANOVA.

## Data Availability

All data generated or analyzed during this study are included in this published article.

## References

[1] Mezzomo N., Maestri B., dos Santos R.L., Maraschin M., Ferreira S.R.S. (2011). Pink Shrimp (*P. brasiliensis* and *P. paulensis*) Residue: Influence of Extraction Method on Carotenoid Concentration. Talanta.

[2] Montero P., Calvo M.M., Gómez-Guillén M.C., Gómez-Estaca J. (2016). Microcapsules Containing Astaxanthin from Shrimp Waste as Potential Food Coloring and Functional Ingredient: Characterization, Stability, and Bioaccessibility. LWT.

[3] Amiguet V.T., Kramp K.L., Mao J., McRae C., Goulah A., Kimpe L.E., Blais J.M., Arnason J.T. (2012). Supercritical Carbon Dioxide Extraction of Polyunsaturated Fatty Acids from Northern Shrimp (Pandalus Borealis Kreyer) Processing by-Products. Food Chem..

[4] Maia M.L., Grosso C., Barroso M.F., Silva A., Delerue-Matos C., Domingues V.F. (2023). Bioactive Compounds of Shrimp Shell Waste from Palaemon Serratus and Palaemon Varians from Portuguese Coast. Antioxidants.

[5] Mezquita P.C., Álvarez C.E., Ramírez J.P., Muñoz W.B., Fuentes F.S., del Carmen Ruiz-Domínguez M. (2020). Isotonic Beverage Pigmented with Water-Dispersible Emulsion from Astaxanthin Oleoresin. Molecules.

[6] Mao X., Guo N., Sun J., Xue C. (2017). Comprehensive Utilization of Shrimp Waste Based on Biotechnological Methods: A Review. J. Clean. Prod..

[7] Gulzar S., Benjakul S. (2018). Ultrasound Waves Increase the Yield and Carotenoid Content of Lipid Extracted From Cephalothorax of Pacific White Shrimp. Eur. J. Lipid Sci. Technol..

[8] Shen Q., Quek S.Y. (2014). Microencapsulation of Astaxanthin with Blends of Milk Protein and Fiber by Spray Drying. J. Food Eng..

[9] Khalid N., Shu G., Kobayashi I., Nakajima M., Barrow C.J. (2017). Formulation and Characterization of Monodisperse O/W Emulsions Encapsulating Astaxanthin Extracts Using Microchannel Emulsification: Insights of Formulation and Stability Evaluation. Colloids Surf. B Biointerfaces.

[10] Fakhri S., Abbaszadeh F., Dargahi L., Jorjani M. (2018). Astaxanthin: A Mechanistic Review on Its Biological Activities and Health Benefits. Pharmacol. Res..

[11] Gulzar S., Raju N., Chandragiri Nagarajarao R., Benjakul S. (2020). Oil and Pigments from Shrimp Processing By-Products: Extraction, Composition, Bioactivities and Its Application—A Review. Trends Food Sci. Technol..

[12] Ambati R., Phang S.-M., Ravi S., Aswathanarayana R. (2014). Astaxanthin: Sources, Extraction, Stability, Biological Activities and Its Commercial Applications—A Review. Mar. Drugs.

[13] Hatta F.A.M., Othman R. (2020). Carotenoids as Potential Biocolorants: A Case Study of Astaxanthin Recovered from Shrimp Waste. Carotenoids: Properties, Processing and Applications.

[14] Herrero M., Cifuentes A., Ibañez E. (2006). Sub- and Supercritical Fluid Extraction of Functional Ingredients from Different Natural Sources: Plants, Food-by-Products, Algae and Microalgae: A Review. Food Chem..

[15] Sachindra N.M., Mahendrakar N.S. (2005). Process Optimization for Extraction of Carotenoids from Shrimp Waste with Vegetable Oils. Bioresour. Technol..

[16] Prayitno D.I., Dewi E.N., Pringgenies D., Brotosudarmo T.H.P. (2022). Green Ultrasound-Assisted Extraction of Astaxanthin from Fermented Rebon Shrimp (Cincalok) Using Vegetable Oils as Solvents. OCL.

[17] Pok P.S., Stefanini M.I., Calvo N.S. (2023). Sustainable Production of Astaxanthin from Dilocarcinus Pagei Crab and Optimisation of Its Extraction with Edible Oils. Heliyon.

[18] Linares G., Rojas M.L. (2022). Ultrasound-Assisted Extraction of Natural Pigments From Food Processing By-Products: A Review. Front. Nutr..

[19] Zou T.-B., Jia Q., Li H.-W., Wang C.-X., Wu H.-F. (2013). Response Surface Methodology for Ultrasound-Assisted Extraction of Astaxanthin from *Haematococcus pluvialis*. Mar Drugs.

[20] Wang X.-S., Wu Y.-F., Dai S.-L., Chen R., Shao Y. (2012). Ultrasound-Assisted Extraction of Geniposide from Gardenia Jasminoides. Ultrason. Sonochem..

[21] Hossain M.B., Brunton N.P., Patras A., Tiwari B., O’Donnell C.P., Martin-Diana A.B., Barry-Ryan C. (2012). Optimization of Ultrasound Assisted Extraction of Antioxidant Compounds from Marjoram (*Origanum majorana* L.) Using Response Surface Methodology. Ultrason. Sonochem..

[22] Santos-Sanchez N.F., Hernández-Carlos B., Torres-Arino A., Salas-Coronado R. (2021). Astaxanthin and Its Formulations as Potent Oxidative Stress Inhibitors. Pharmacogn. Rev..

[23] Martínez-Delgado A.A., Khandual S., Villanueva–Rodríguez S.J. (2017). Chemical Stability of Astaxanthin Integrated into a Food Matrix: Effects of Food Processing and Methods for Preservation. Food Chem..

[24] Liu X., McClements D.J., Cao Y., Xiao H. (2016). Chemical and Physical Stability of Astaxanthin-Enriched Emulsion-Based Delivery Systems. Food Biophys..

[25] Boonlao N., Shrestha S., Sadiq M.B., Anal A.K. (2020). Influence of Whey Protein-Xanthan Gum Stabilized Emulsion on Stability and in Vitro Digestibility of Encapsulated Astaxanthin. J. Food Eng..

[26] Gallardo G., Guida L., Martinez V., López M.C., Bernhardt D., Blasco R., Pedroza-Islas R., Hermida L.G. (2013). Microencapsulation of Linseed Oil by Spray Drying for Functional Food Application. Food Res. Int..

[27] Phadtare I., Vaidya H., Hawboldt K., Cheema S.K. (2021). Shrimp Oil Extracted from Shrimp Processing By-Product Is a Rich Source of Omega-3 Fatty Acids and Astaxanthin-Esters, and Reveals Potential Anti-Adipogenic Effects in 3T3-L1 Adipocytes. Mar. Drugs.

[28] Aneesh P.A., Anandan R., Kumar L.R.G., Ajeeshkumar K.K., Kumar K.A., Mathew S. (2023). A Step to Shell Biorefinery—Extraction of Astaxanthin-Rich Oil, Protein, Chitin, and Chitosan from Shrimp Processing Waste. Biomass Convers. Biorefin..

[29] Sánchez-Camargo A.P., Meireles M.Â.A., Ferreira A.L.K., Saito E., Cabral F.A. (2012). Extraction of ω-3 Fatty Acids and Astaxanthin from Brazilian Redspotted Shrimp Waste Using Supercritical CO_2_ + Ethanol Mixtures. J. Supercrit. Fluids.

[30] Christaki E., Bonos E., Giannenas I., Florou-Paneri P. (2013). Functional Properties of Carotenoids Originating from Algae. J. Sci. Food Agric..

[31] Oh S., Kim Y.J., Lee E.K., Park S.W., Yu H.G. (2020). Antioxidative Effects of Ascorbic Acid and Astaxanthin on ARPE-19 Cells in an Oxidative Stress Model. Antioxidants.

[32] Igielska-Kalwat J., Gościańska J., Nowak I. (2015). Carotenoids as Natural Antioxidants. Postep. Hig. Med. Dosw..

[33] Sharayei P., Azarpazhooh E., Zomorodi S., Einafshar S., Ramaswamy H.S. (2021). Optimization of Ultrasonic-Assisted Extraction of Astaxanthin from Green Tiger (*Penaeus semisulcatus*) Shrimp Shell. Ultrason. Sonochem..

[34] Li Y., Fabiano-Tixier A.S., Chemat F. (2017). Vegetable Oils as Alternative Solvents for Green Extraction of Natural Products. Edible Oils: Extraction, Processing, and Applications. Edible Oils: Extraction, Processing, and Applications.

[35] Calligaris S., Mirolo G., Da Pieve S., Arrighetti G., Nicoli M.C. (2014). Effect of Oil Type on Formation, Structure and Thermal Properties of γ-Oryzanol and β-Sitosterol-Based Organogels. Food Biophys..

[36] Challis R.E., Pinfield V.J. (2014). Ultrasonic Wave Propagation in Concentrated Slurries—The Modelling Problem. Ultrasonics.

[37] Kang C.D., Sim S.J. (2008). Direct Extraction of Astaxanthin from Haematococcus Culture Using Vegetable Oils. Biotechnol. Lett..

[38] El-Bialy H.A.A., El-Khalek H.H.A. (2020). A Comparative Study on Astaxanthin Recovery from Shrimp Wastes Using Lactic Fermentation and Green Solvents:An Applied Model on Minced Tilapia. J. Radiat. Res. Appl. Sci..

[39] Orsavova J., Misurcova L., Ambrozova J., Vicha R., Mlcek J. (2015). Fatty Acids Composition of Vegetable Oils and Its Contribution to Dietary Energy Intake and Dependence of Cardiovascular Mortality on Dietary Intake of Fatty Acids. Int. J. Mol. Sci..

[40] Bakre S.M., Gadmale D.K., Toche R.B., Gaikwad V.B. (2015). Rapid Determination of Alpha Tocopherol in Olive Oil Adulterated with Sunflower Oil by Reversed Phase High-Performance Liquid Chromatography. J. Food Sci. Technol..

[41] Etoh H., Suhara M., Tokuyama S., Kato H., Nakahigashi R., Maejima Y., Ishikura M., Terada Y., Maoka T. (2012). Auto-Oxidation Products of Astaxanthin. J. Oleo Sci..

[42] Kamezaki C., Nakashima A., Yamada A., Uenishi S., Ishibashi H., Shibuya N., Hama S., Hosoi S., Yamashita E., Kogure K. (2016). Synergistic Antioxidative Effect of Astaxanthin and Tocotrienol by Co-Encapsulated in Liposomes. J. Clin. Biochem. Nutr..

[43] Xie L., Ciftci O., Zhang Y. (2020). Encapsulation of Astaxanthin-Enriched Camelina Oil Extract in Ovalbumin/Gum Arabic Stabilized Emulsion with/without Crosslinking by Tannic Acid. ES Food Agrofor..

[44] Gomez-Estaca J., Comunian T.A., Montero P., Ferro-Furtado R., Favaro-Trindade C.S. (2016). Encapsulation of an Astaxanthin-Containing Lipid Extract from Shrimp Waste by Complex Coacervation Using a Novel Gelatin–Cashew Gum Complex. Food Hydrocoll..

[45] Silva A., Rodrigues B., Silva L., Rodrigues A. (2018). Drying and Extraction of Astaxanthin from Pink Shrimp Waste (*Farfantepenaeus subtilis*): The Applicability of Spouted Beds. Food Sci. Technol..

[46] Bligh E.G., Dyer W.J. (1959). A Rapid Method of Total Lipid Extraction and Purification. Can. J. Biochem. Physiol..

[47] Nasopoulou C., Stamatakis G., Demopoulos C.A., Zabetakis I. (2011). Effects of Olive Pomace and Olive Pomace Oil on Growth Performance, Fatty Acid Composition and Cardio Protective Properties of Gilthead Sea Bream (*Sparus aurata*) and Sea Bass (*Dicentrarchus labrax*). Food Chem..

[48] Nasopoulou C., Demopoulos C.A., Zabetakis I. (2012). Effect of Freezing on Quality of Sea Bass and Gilthead Sea Bream. Eur. J. Lipid Sci. Technol..

[49] Michalaki A., Karantonis H.C., Kritikou A.S., Thomaidis N.S., Dasenaki M.E. (2023). Ultrasound-Assisted Extraction of Specific Phenolic Compounds from Petroselinum Crispum Leaves Using Response Surface Methodology and HPLC-PDA and Q-TOF-MS/MS Identification. Appl. Sci..

[50] Rahmalia W., Dasilia C., Usman T., Prayitno D.I., Nurbaeti S.N. (2022). Astaxanthin and Omega-3-Rich Oil from Fermented Acetes (Cincalok) and Its Application as Bioactive Additive and Sunscreen in Lotion. OCL.

[51] Haniff M., Yahaya S.A., Aziz N.S., Wan Mustapha W.A., Sofian-Seng N., Rahman H.A., Mohd Razali N.S., Lim S.J. (2020). Development of Carotenoid-rich Mayonnaise Using Carotino Oil. J. Food Process Preserv..

